# Discovery of stripe rust resistance with incomplete dominance in wild emmer wheat using bulked segregant analysis sequencing

**DOI:** 10.1038/s42003-022-03773-3

**Published:** 2022-08-17

**Authors:** Valentyna Klymiuk, Harmeet Singh Chawla, Krystalee Wiebe, Jennifer Ens, Andrii Fatiukha, Liubov Govta, Tzion Fahima, Curtis J. Pozniak

**Affiliations:** 1 Crop Development Centre and Department of Plant Sciences, University of Saskatchewan, Saskatoon, SK S7N 5A8 Canada; 2 Institute of Evolution, University of Haifa, 199 Abba-Hushi Avenue, Mt. Carmel, 3498838 Haifa, Israel; 3 Department of Evolutionary and Environmental Biology, University of Haifa, 199 Abba-Hushi Avenue, Mt. Carmel, 3498838 Haifa, Israel

**Keywords:** Plant genetics, Plant breeding, Genome informatics

## Abstract

Durable crop disease resistance is an essential component of global food security. Continuous pathogen evolution leads to a breakdown of resistance and there is a pressing need to characterize new resistance genes for use in plant breeding. Here we identified an accession of wild emmer wheat (*Triticum turgidum* ssp. *dicoccoides*), PI 487260, that is highly resistant to multiple stripe rust isolates. Genetic analysis revealed resistance was conferred by a single, incompletely dominant gene designated as *Yr84*. Through bulked segregant analysis sequencing (BSA-Seq) we identified a 52.7 Mb resistance-associated interval on chromosome 1BS. Detected variants were used to design genetic markers for recombinant screening, further refining the interval of *Yr84* to a 2.3–3.3 Mb in tetraploid wheat genomes. This interval contains 34 candidate genes encoding for protein domains involved in disease resistance responses. Furthermore, KASP markers closely-linked to *Yr84* were developed to facilitate marker-assisted selection for rust resistance breeding.

## Abstract (web summary)

Stripe rust is an epidemic disease of wheat that can cause major economic losses. A resistant allele is identified in wild emmer wheat using a BSA-seq approach.

## Introduction

Wheat (*Triticum* spp.) is one of the most important and widely grown cereal crops in the world providing ~20% of calories for global consumption (Food and Agriculture Organization Corporate Statistical Database (FAOSTAT) (https://www.fao.org/faostat/). Bread and other bakery products, pasta, couscous, bulgur, freekeh, and other food commodities are made from wheat. In addition, wheat is a source of animal feed, and its grain is used by industry to produce malt, starch, gluten, dextrose, and alcohol. Wheat production is stagnating and there is a need to increase yields, as well as reduce the yield gap (the differential between genetic potential achieved by breeding and actual production potential). Wheat production and quality is negatively impacted by biotic (disease and pest) stresses, of which fungal diseases represent the predominant threat to wheat with at least 25 pathogens known to cause diseases^1^.

The biotrophic fungus *Puccinia striiformis* f. sp. *tritici* (*Pst*) is the causal agent of wheat stripe (yellow) rust. Stripe rust epidemics are widespread in all wheat-producing areas with over 85% of the world’s wheat production impacted by infection^2^. Average yield losses per year range from 5–10%, but 100% yield losses can occur in cases of early infection of highly susceptible varieties^3^. Growing genetically resistant wheat cultivars is the most environmentally sustainable and cost-effective strategy to control stripe rust disease. To date, 130 *Pst* resistance genes (*Yr* genes) have been reported^4^. The majority of these are inherited as dominant genes, while only a few are recessive or incompletely (partially) dominant. Plant-pathogen co-evolution can result in new pathogen strains that acquire structural modifications within target avirulence proteins that are typically recognized by wheat resistance proteins. An absence of this recognition allows the *Pst* fungus to successfully colonize plant tissues. As a consequence, many *Yr* genes are no longer effective. Hence, there is a need for continuous identification and characterization of novel sources of resistance.

While several *Yr* genes have been genetically localized, very few have been cloned and functionally validated^4^. To date, only eight *Yr* genes have been functionally validated: *Yr36*^5^, *Yr18*(*Lr34)*^6^, *Yr46*(*Lr67)*^7^, *Yr15*^8^, *Yr5*/*YrSp*^9^, *Yr7*^9^, *YrAS2388*^10^, and *YrU1*^11^. Although *Yr10* was provisionally cloned (*Yr10*_*CG*_, GenBank: AF149112)^12^, later studies determined that the *Yr10* localized 1.2 cM distal to *Yr10*_*CG*_^13^. With recent advancements in genomic resources and the availability of high-throughput genotyping platforms, novel methods for gene discovery, mapping and cloning are becoming available. Bulked segregant analysis (BSA) was first described in 1991^14,15^. Classical BSA experiments generate genotypes from a bi-parental population that is segregating for the trait of interest. DNA from phenotypically contrasting individuals (e.g., resistant vs. susceptible) are pooled into two separate bulks and are independently genotyped using available low-density molecular markers. In the case of additive gene action, all unlinked markers will be heterozygous, whereas markers linked to the trait will be (near) homozygous. A chromosomal region enriched for homozygous markers represents the likely location for the gene under study. However, the degree of homozygosity enrichment will vary depending on the type of gene action (additive vs. non-additive) and/or the type and structure of the mapping population. Whole-genome next generation sequencing technologies provide high-density variant calls representing single nucleotide polymorphisms (SNPs) and indels (insertions/deletions) that can easily be applied to BSA. This simple BSA-Seq strategy is effective to identify a mapping interval and associated SNPs and has been successfully applied to identify genomic loci controlling key agronomic traits, such as 100-grain weight in chickpea^16,17^, flowering time in cucumber^18^, fruit weight in tomato^19^, and anthocyanin synthesis in *Brassica rapa*^20^.

Genetically, wheat is an allopolyploid with a complex evolutionary history involving a set of hybridization events between diploid and tetraploid species. Cultivated wheat includes two main species: tetraploid durum wheat (*Triticum turgidum* ssp. *durum;* 2*n* = 4*x* = 28) and hexaploid bread wheat (*T. aestivum;* 2*n* = 6*x* = 42). Diploid and tetraploid wheat progenitors still exist in the wild and can be used to enrich the cultivated wheat gene pool with novel genes or alleles. Nearly half of the cloned *Yr* genes, i.e., *Yr36*, *Yr15*, *YrAS2388* and *YrU1*, have wild wheat origins, including from tetraploid wild emmer wheat (*T. turgidum* ssp. *dicoccoides*) or the diploid wheats (2*n* = 2*x* = 14) *T. urartu* and *Aegilops tauschii*. Traditionally, the large highly repetitive genomes of wheat have hampered gene discovery efforts^21^, but several high-quality reference genome assemblies have become available recently for diploid^22^, tetraploid^23,24^, and hexaploid wheat species^21,25^. These assemblies provide an unprecedented opportunity to support rapid discovery and characterization of novel resistance genes^26^.

In the current study, we identified a wild emmer wheat accession, PI 487260, that is highly resistant to stripe rust. We applied an advanced BSA-Seq approach to map a single gene *Yr84*, to the short arm of chromosome 1B. Further mapping and phenotypic analysis of homozygous recombinant lines allowed us to narrow the physical region associated with resistance and to suggest candidate genes. Precise investigation of the phenotypic responses within genetically diverse populations combined with genotypic data, allowed us to uncover the incomplete dominance nature of *Yr84*. The use of *Yr84* in wheat breeding programs could be facilitated by marker-assisted selection utilizing the closely linked KASP makers developed in this study.

## Results

### PI 487260 carries a single resistance gene effective against multiple isolates of stripe rust

**Fig. 1:**
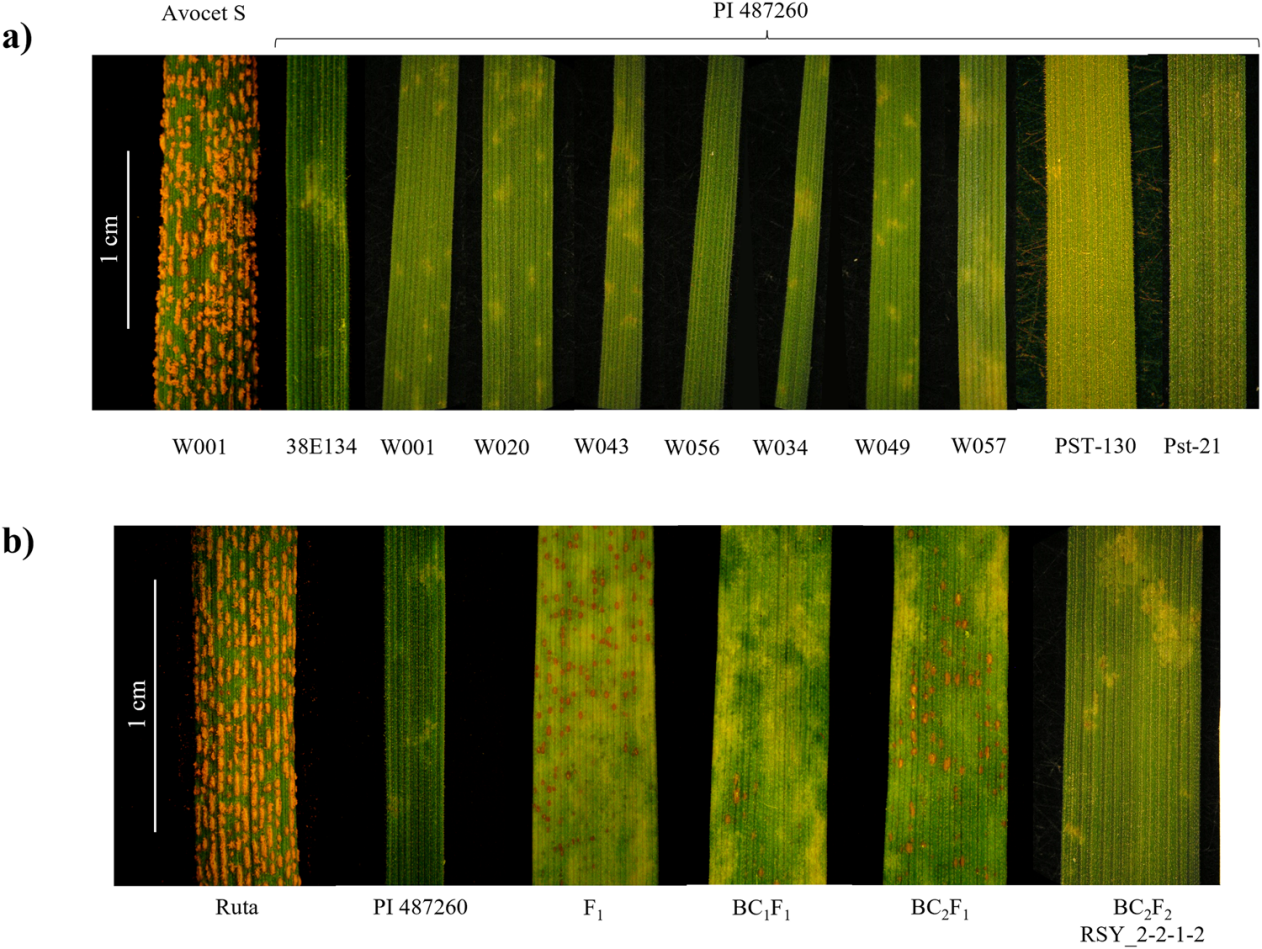
Phenotypic responses of *Yr84* at wild emmer wheat donor line PI 487260 and at different stages of its introgression into cultivated wheat backgrounds to inoculation with stripe rust. **a** PI 487260 response to inoculation with ten *Pst* isolates from Israel, Canada and the USA. **b** Reactions to *Pst* race W001 observed at multiple filial generations of *Yr84* introgression into hexaploid bread wheat cv. Ruta. Full resistance is expressed only after *Yr84* is fixed in homozygous state at BC_2_F_2_ and following generations.

In seedling tests, the wild emmer wheat accession PI 487260 expressed complete resistance (Infection Type (IT) = 0–2) to ten *Pst* isolates collected from Canada, the USA and Israel (Fig. 1a). We crossed PI 487260 with the susceptible durum wheat cv. Kronos to develop the KSY_10 F_2_ mapping population. The population exhibited a 3:1 resistant (R) + moderate resistant (MR):susceptible (S) phenotypic segregation ratio in response to *Pst* race W001 inoculation (*χ*^2^ = 2.84; *p* = 0.05), suggesting the presence of a single resistance gene, which we designated as *Yr84* as per recommended rules for gene symbolization in wheat (https://wheat.pw.usda.gov/GG3/wgc).

### BSA-Seq of PI 487260-derived stripe rust resistance

To rapidly identify the genomic region(s) associated with stripe rust resistance, we applied a BSA-Seq approach. In total, 92 F_2_ plants of KSY_10 population were phenotyped at the seedling stage following inoculation with the *Pst* race W001. From these, 26 of the most resistant plants (IT = 1–4) and 26 susceptible plants (IT = 9) were selected, and indexed DNA libraries were prepared for each F_2_ plant separately. While non-indexed DNA pools are typical for BSA-Seq experiments, we decided to indexed each of the libraries given the range of IT response in the resistant bulk, and the possibility of heterozygous plants being chosen for the DNA bulk^27^. These DNA libraries were pooled to form the R- and S-bulks and used for Illumina paired-end sequencing. The resistant (PI 487260) and susceptible (Kronos) parents were added to their corresponding bulk.

**Fig. 2:**
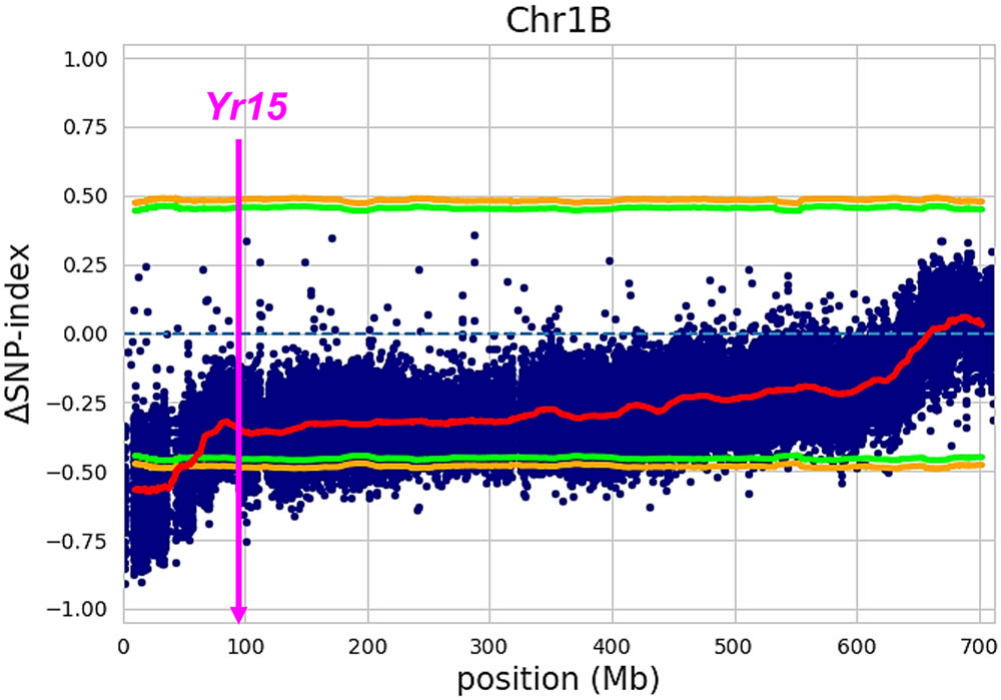
Graphical representation of the results of BSA-Seq analysis for chromosome 1B with enrichment in the 0-52.7 Mb region. Each blue dot corresponds to a SNP between the DNA bulks. The red line indicates the mean ΔSNP-index. Orange and green lines represent coverage-adjusted confidence interval of the simulated ΔSNP-index at 99% and 95%, respectively. The physical position of the cloned stripe rust resistance gene *Yr15* is indicated.

An average of ~200 M paired-end reads (150 bp) per genotype (range: 69,486,314–365,334,783) were generated resulting in a total of ~5600 M reads for the S-bulk and ~5100 M reads for the R-bulk. The average genome coverage (based on an estimated genome size of 11 Gb) per genotype used for downstream analyses for the R- and S- bulks was 5.2X and 5.7X, respectively. The resulting short reads from each bulk were mapped to the annotated Zavitan WEWSeq v2.0 reference assembly^24^, with 97.3 and 97.5% of the reads mapping for the R- and S-bulk, respectively. In total 1,345,590 SNPs were identified between the two bulks. To identify the chromosomal regions associated with enrichment, we calculated the ΔSNP-index between R- and S-bulks in a 20,000 bp sliding windows (with a step size of 100 kbp; Supplementary Data 1) and scanned for regions of genome enriched (Supplementary Figs. 1 and 2). We identified 4889 SNPs/indels (Supplementary Data 2) and detected a single peak of variant enrichment spanning a 52.7 Mb interval on chromosome 1B (0 to 52.7 Mb) that passed the coverage-adjusted confidence interval at the 99% threshold (Fig. 2; Supplementary Figs. 1 and 2). We also identified smaller peaks of enrichment on chromosomes 2B, 3A, 4B and 5B, but these did not pass the significance threshold (*p* > 0.05).

### The physical localization of *Yr84*

**Fig. 3:**
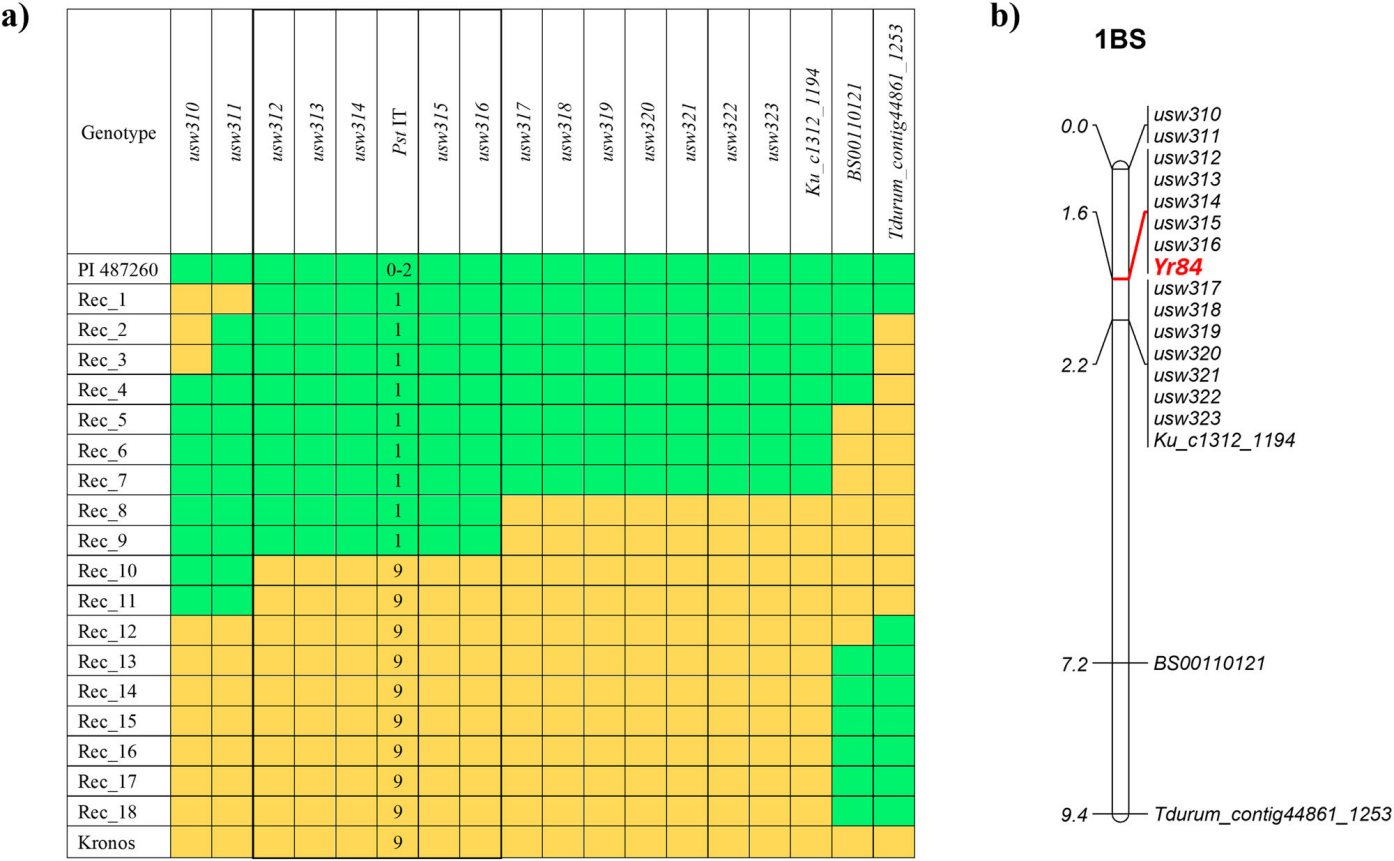
Genetic position of *Yr84* on chromosome arm 1BS. **a** Recombinant haplotypes in chromosome 1B region of *Yr84*. Marker alleles are as following: green = identical to allele of the *Yr84* carrier PI 487260; yellow = identical to allele of the non-carrier Kronos. Phenotyping reaction to *Pst* W001 is based on the 0-9 IT scale: resistance is marked in green; susceptibility is marked in yellow. **b** The genetic map (cM) is based on 92 F_2_ plants from the KSY_10 population (PI 487260 × Kronos).

In order to more precisely localize *Yr84*, we used variant calls from the 0–52.7 Mb interval to develop KASP markers for mapping. First, we converted the markers *Ku_c1312_1194*, *BS00110121*, and *Tdurum_contig44861_1253* from the available 90 K SNP array^28^ that were physically mapped to chromosome 1B at 14.6, 25.8 and 30.9 Mb positions, respectively, in the Zavitan assembly. The 92 F_2_ KSY_10 plants used to select resistant and susceptible plants for BSA-Seq bulks were genotyped with these markers. These results, combined with phenotypic data for all 92 F_2_ plants, indicated that *Yr84* is localized distal to these three markers (Fig. 3b). The additional markers developed from BSA-Seq (*usw310*-*usw326*) detected 18 recombinant plants (Rec_1 to Rec_18). These were self-pollinated to develop homozygous F_3_ plants that were phenotyped and genotyped for precise haplotype analysis (Fig. 3a). Following this analysis, the physical position of *Yr84* was reduced to a 2.2 cM genetic region (Fig. 3b). The *usw310* and *usw311* markers were located 1.6 cM distal to *Yr84*, while *usw317*-*usw323* and *Ku_c1312_1194* markers mapped 0.8 cM proximal to *Yr84* (Fig. 3b). The *Yr84* locus co-segregated with the markers *usw312*-*usw316* (Fig. 3b). Haplotype analysis allowed us to narrow the location of *Yr84 * to a 2.33 Mb physical interval in the Zavitan reference genome assembly (9.66–11.99 Mb on Chr1B) and a 3.37 Mb physical interval in the durum wheat Svevo (RefSeq Rel. 1.0) reference assembly^23^ (4.91–8.28 Mb on Chr1B) (Supplementary Table 1).

### *Yr84* is different from previously characterized *Yr* genes

Several genes were previously localized to chromosome 1BS and of these, only the genetic position of  *YrAlp* was congruent with that of *Yr84*. We performed a haplotype analysis using markers from the mapped interval of *Yr84*, which revealed the uniqueness of PI 487260 haplotype compared with bread wheat cv. Alpowa (*YrAlp* carrier) and control samples (Supplementary Fig. 3). Taken together, these results support that *Yr84* is not allelic to *YrAlp*.

Among wheat stripe rust resistance genes that have been cloned, only *Yr15* localizes to the short arm of chromosome 1B. Our BSA-Seq results show that the physical position of *Yr15* was >45 Mb from *Yr84* (Fig. 2). Moreover, after mapping with KASP markers, *Yr84* localized ~86 Mb from *Yr15* (Supplementary Table 1). In addition, haplotype analysis showed that the *Yr15* carrier Avocet+*Yr15* is distinct from PI 487260 in the *Yr84* region (Supplementary Fig. 3). We also confirmed the absence of the *Yr15* functional allele (*Wtk1*) in the PI 487260 background using the two functional *Yr15* KASP markers^29^ (Supplementary Fig. 4).

In addition, we performed haplotype analysis for Avocet *Yr* differential lines carrying single genes *Yr1*, *Yr5*, *Yr6*, *Yr7*, *Yr8*, *Yr9*, *Yr10*, *Yr17*, *Yr18*, *Yr24*, *Yr26*, *Yr27*, *Yr32*, *YrSp*, *YrA* and confirmed that none shared the PI 487260 haplotype in the *Yr84* region (Supplementary Fig. 3).  Taken together, these results support that *Yr84* is unique from these previously characterized genes.

### *Yr84* is an incompletely dominant gene

**Fig. 4:**
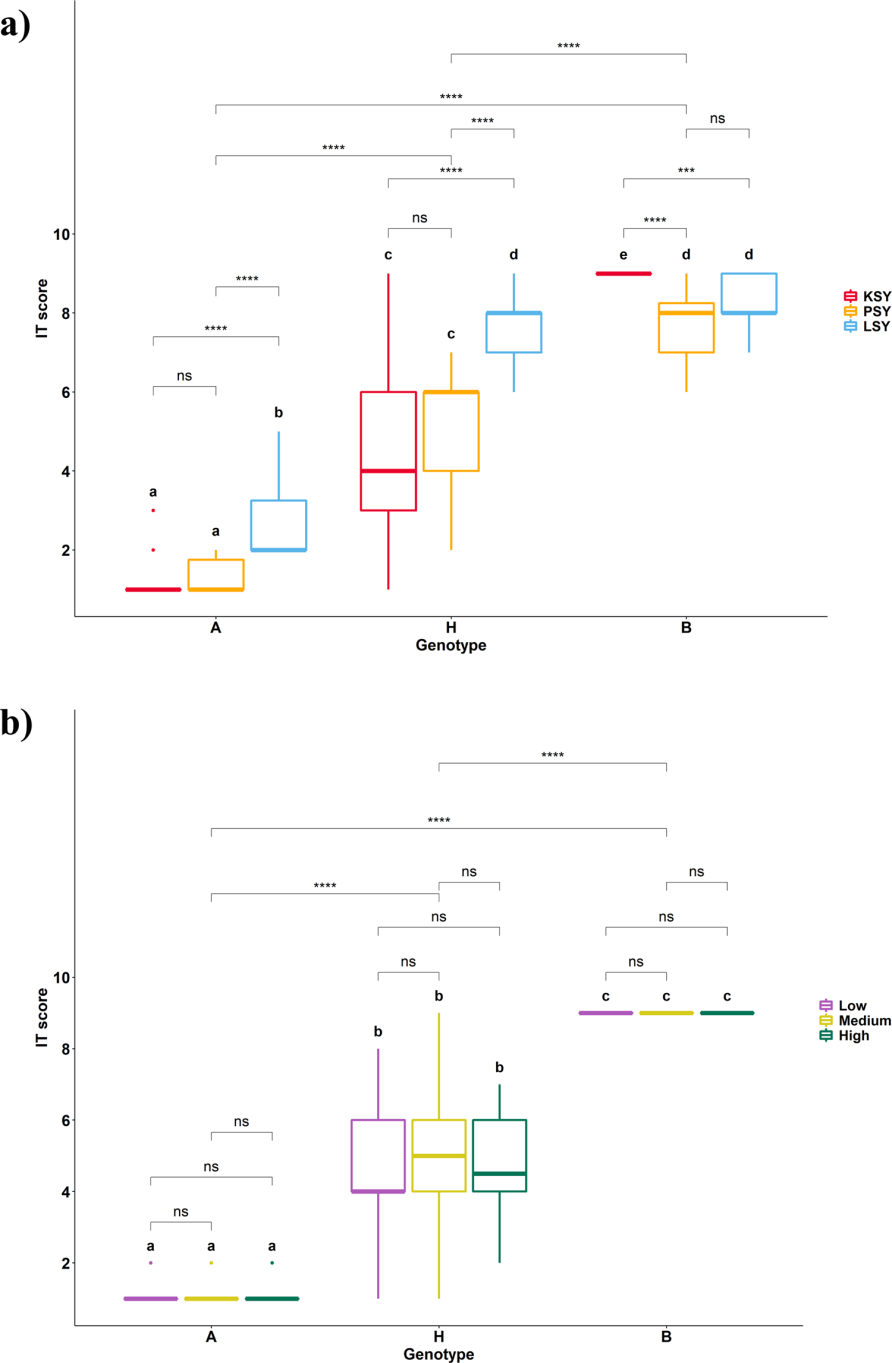
Boxplots of IT score and results of multiple means comparison using the Tukey–Kramer post hoc test between genotypes *A*, *H* and *B*. **a** Analysis for three *Yr84* populations and **b** for three treatments of KSY population with low, medium, and high *Pst* spore concentrations. Population codes are as following: KSY – PI 487260 × Kronos; PSY – KSY_10-64(*Yr84* carrier) × CDC Precision; LSY – RSY_2-2-1-2(*Yr84* carrier) × CDC Landmark. Boxplot shows the median (middle quartile), interquartile range (IQR) from 25th to the 75th percentile, “minimum” whisker as Q1 – 1.5*IQR and “maximum” whisker as Q3 + 1.5*IQR. Letters under each boxplot represent significantly different groups of IT score (two tailed; *p* < 0.05). Significance codes: **** < 0.0001; 0.0001 < *** < 0.001; 0.001 < ** < 0.01; 0.01 < * < 0.05; ns > 0.05.

A combination of phenotypic and genotypic data (using the *usw314* marker) for 92 F_2_ plants from the KSY_10 population confirmed that *Yr84* was incompletely dominant. The presence of the homozygous *Yr84* (genotype *A*, hereafter) resulted in plants that expressed complete resistance (IT = 1–2), while those plants with the heterozygous *Yr84* (genotype *H*, hereafter) presented a wide range of phenotypic responses (IT = 1–9) (Fig. 4a). These results were confirmed with 30 F_1_ plants from the same PI 487260 × Kronos cross, for which resistance responses varied in a range IT = 1–7.

We generated two additional F_2_ populations to confirm the incomplete dominance nature of *Yr84*—a tetraploid F_2_ population designated as PSY (KSY_10-64(*Yr84* carrier)×CDC Precision) and a hexaploid F_2_ population named LSY (RSY_2-2-1-2 (Fig. 1b) (*Yr84* carrier) × CDC Landmark). Phenotypic responses to stripe rust differed between populations (*p* < 0.0001; Supplementary Table 2 and Fig. 4a) and both populations showed variable resistance response depending on the zygosity state of *Yr84* (*p* < 0.0001; Fig. 4a; Supplementary Data 3). Population comparisons showed no significant differences between the means of the tetraploid populations KSY and PSY harboring genotypes *A* or *H*, while the hexaploid population LSY differed significantly from both tetraploid populations (*p* < 0.0001). At the same time, *Yr84* non-carrier genotypes (genotype *B*, hereafter) differed significantly between KSY and PSY (*p* < 0.0001) perhaps due to the effect of additional resistance gene(s) in CDC Precision (Supplementary Table 3; Fig. 4a). Interestingly, the F_2_ plants with the genotype *H* of *Yr84* in the hexaploid population LSY were highly susceptible (mean IT = 7.6) and were not significantly different from genotypes not carrying *Yr84* (genotype *B*) (Supplementary Table 3). Introgression of *Yr84* into a second hexaploid background (cv. Ruta) showed similar results: heterozygous F_1_, BC_1_F_1_, and BC_2_F_1_ plants exhibited partial resistance to *Pst* inoculation, and only homozygous BC_2_F_2_ plants and the following generations were fully resistant (Fig. 1b).

To rule out the possibility that incomplete dominance was a consequence of spore load, we performed inoculations with high, medium, or low concentrations of spores and evaluated phenotypic response. Varying spore concentration did not influence phenotypic expression of *Yr84* resistance in the KSY F_2_ population (Fig. 4b and Supplementary Table 4). The effect of the genotype and pairwise-comparisons between the associated genotypic groups were highly significant (*p* < 0.0001) (Fig. 4b; Supplementary Table 4 and Supplementary Data 4). Results from these experiments also re-affirmed that genotypes with either the homozygous genotype (*A* or *B*) showed narrow distributions of IT, while genotypes with the heterozygous genotype (*H*) had a wide range of IT scores.

### Candidate genes for *Yr84*

We used the 2.33 Mb interval from Zavitan and 3.37 Mb interval in Svevo identified from our haplotype analysis to extract lists of annotated genes from both assemblies. Combined, this included 79 genes (Supplementary Table 1), of which 47 genes were found in both annotations, 14 genes were unique to wild emmer wheat Zavitan (in underlined font in Supplementary Table 1), and 18 were specific to Svevo (in italic font in Supplementary Table 1). A manual, conserved domain search revealed that 34 of the 79 genes encode for protein domains known to be involved in disease resistance responses (in bold font in Supplementary Table 1), such as the NB-ARC super family, protein kinases, Rx_N domain, leucine-rich repeat receptor-like protein kinase, Dirigent super family and the LRR super family. These genes represent reasonable candidate genes for *Yr84* that could be prioritized for functional validation.

## Discussion

The purpose of the current study was to precisely map the gene underlying the effective stripe rust resistance we identified from the wild emmer wheat accession PI 487260. To accomplish this, we applied a BSA-Seq approach to a well phenotyped bi-parental F_2_ population, for rapid genomic localization of resistance locus to chromosome 1B. Wheat is a polypoid species, and its genome is highly repetitive^25^. This can complicate identification of causal genes underlying a desirable phenotype. To reduce the influence of genome complexity, others have used a combination of BSA with RNA-Seq^26^ or Exome Capture^30^ sequencing to fine-map/clone agronomically important genes. Using an RNA-Seq approach has limitations because it requires that the underlying gene is expressed at the time of sample collection. In contrast, we used a whole-genome sequencing approach, that can be applied  to wheat  utilizing available analysis pipelines^27^. One of the major advantages of BSA-Seq over traditional linkage mapping is that it can rapidly identify a major chromosomal location of genetic loci underlying traits, without the need to genotype an entire population. The only requirement for BSA-Seq is that the trait segregates sufficiently into two distinct phenotypes and a suitable reference assembly to which sequenced reads can be aligned. However, the latter is no longer an issue with wheat given the increasing number of wheat genome assemblies that are now available^21–25^. Compared to SNP arrays, BSA-Seq based on next generation sequencing also provides ultra-high SNP density in genomic regions of interest that can then be used to develop markers for fine mapping. Indeed, the high-density SNPs that we detected in the current study were paramount to develop robust markers to support precise localization of *Yr84*. This additional resolution was necessary for the identification of the candidate genes of which 34 could be prioritized for functional validation as they encode for protein domains known to be involved in disease resistance responses.

A number of wheat *Yr* genes including *Yr15*/*YrH52*/*YrG303*^8,31^, *Yr24*/*Yr26*^32^, *Yr10*^13^, *YrC142*^33^, *YrAlp*^34^, *Yr64*^35^, and *Yr65*^35^, have been previously mapped on chromosome arm 1BS. Many genome-wide association studies have also shown marker-trait associations for stripe rust resistance on 1BS that could overlap with the positions of these *Yr* loci^36–44^. Of these genes, only *Yr15*/*YrH52*/*YrG303* has been cloned as *Wheat Tandem Kinase 1* (*WTK1*) and here we show that *Yr84* localizes ∼86 Mb distal to *WTK1*, and that PI 487260 lacks *Wtk1* based on genotyping with functional markers. The *Yr24*/*Yr26*, *YrC142*, *Yr64*, and *Yr65* genes all localize proximal to *Yr15* and, therefore, are not *Yr84*^33,35^. The physical position of *Yr10*^13^ is distal to *Yr84*. The most proximal candidate gene for *Yr10* (TRIDC1BG000420 at position 1397243–1402456^13^) and the most distal candidate gene for *Yr84* (TRIDC1BG001810 at position 9657221-9658930 (Supplementary Table 1) are ~8.3 Mb apart, suggesting that these are indeed two different genes. Furthermore, *Yr10* is a race-specific gene, and five out of the ten *Pst* races/isolates used in the current study to which PI 487260 was resistant, are virulent on *Yr10* (Supplementary Table 5), indicating different race specificities for *Yr84* and *Yr10*. We also show that Avocet *Yr* differential lines (*Yr1*, *Yr5*, *Yr6*, *Yr7*, *Yr8*, *Yr9*, *Yr10*, *Yr17*, *Yr18*, *Yr24*, *Yr26*, *Yr27*, *Yr32*, *YrSp*, *YrA*) do not share the PI 487260 haplotype in the *Yr84* region (Supplementary Fig. 3).

The race-specific, partially dominant gene *YrAlp* has been localized previously to chromosome 1BS and its genetic position overlaps with that of *Yr84*^34^. Unfortunately, the sets of *Pst* races used in the *YrAlp* study^34^ and our study have little overlap (only Pst-21 was used in both studies and it was avirulent on both genes) and, therefore, we cannot confirm allelism based on comparison of their race specificities. On the other hand, haplotype analysis revealed that the PI 487260 haplotype is unique from that of Alpowa, suggesting that *Yr84* is not allelic to *YrAlp* (Supplementary Fig. 3). Also, *Yr84* originates from tetraploid wild emmer wheat, while *YrAlp* was mapped in a hexaploid bread wheat cv. Alpowa, which does not appear to contain wild emmer wheat in its pedigree (http://wheatpedigree.net/).

In three populations (tetra- and hexaploid), *Yr84* exhibited incomplete dominance where plants homozygous for *Yr84* expressed greater resistance than those in the heterozygous condition. Both tetraploid populations had similar responses to *Pst* inoculation, while the hexaploid population was generally more susceptible compared to the tetraploid populations. This later observation could be the result of hexaploid D genome-derived suppression of resistance^45^ or that *Yr84* resistance is background-dependent, as was previously shown for other resistance genes^31^. A number of genes conferring resistance to bacterial, viral and biotrophic fungal pathogens in various plant species were previously reported to be incompletely dominant. These include the *Pto* locus providing resistance to tomato bacterial speck disease^46^, tomato *Cf* genes for resistance to fungal pathogen *Cladosporum fulvum*^47^, *Ty-6* gene effective against monopartite tomato yellow leaf curl virus (TYLCV) and bipartite tomato mottle virus (ToMoV)^48^, *Arabidopsis RPP4* gene conferring downy mildew resistance^49^, and  some wheat and barely rust resistance genes^50^.

Several possible mechanisms may explain the incomplete dominance of resistance we observed in our study. First it may be related to a greater proportion of functional resistance protein in homozygous vs. heterozygous individuals resulting from higher expression levels and/or post-translational modifications leading to activation. For example, it has been suggested that two copies of the incompletely dominant resistance gene *Cf*  in homozygous state result in greater amounts of active protein, while in heterozygous individuals, lower levels are not sufficient to activate the plant’s defense responses^47^. Secondly, it is also possible that resistance proteins function as part of a resistance complex, such that higher expression in homozygous individuals increases the availability of functional proteins to initiate complex formation and an associated resistance response. For example, Pto kinase proteins function as part of oligomeric immune complex with Prf NLR proteins for effector recognition^51^. The presence of oligomerized pre-formed complexes (resistomes) is not uncommon and is well documented for Arabidopsis ZAR^52–54^, ROQ1^55^, and RPP1^56^ immune systems. The precise 3D formation of resistomes is crucial for their function as cation channels to trigger plant immunity and cell death^57^. It is possible that a non-functional resistance protein copy in a heterozygous plant disrupts formation of the resistome complex, resulting in partial functionality. Taken together, a limitation of functional resistance proteins and/or the formation of required resistance complexes in heterozygous individuals could explain the incomplete dominance of *Yr84*. However, cloning the casual gene and its functional characterization are necessary to confirm this hypothesis.

The current study demonstrates the importance of crop wild relative gene pool as a source for novel genes and alleles that can be used for crop resistance breeding^58^. We showed that *Yr84*, when in a homozygous state can provide full resistance when introgressed into cultivated wheat backgrounds: tetraploid durum wheat cv. Kronos, CDC Precision and hexaploid bread wheat cv. Ruta, CDC Landmark. To facilitate incorporation of *Yr84* into wheat improvement programs and selection for its homozygous state, we have developed closely linked, codominant KASP markers that we have validated in a collection of durum and bread wheat lines as being sufficiently robust for marker-assisted selection (Supplementary Fig. 3). These markers can be used for pyramiding of multiple stripe rust resistance genes (for example with *Yr15*) into a single cultivar to provide durable disease protection and to facilitate sustainable farming.

## Methods

### Plant and fungal material

Wild emmer wheat accession PI 487260 (SY 20121) was originally collected at As Suwaydā’, Syria (32.6478, 36.7900) at 1530 meters above mean sea level (MASL) in 1980 (https://npgsweb.ars-grin.gov/gringlobal/accessiondetail?id=1382196). Seeds of this accession and bread wheat cv. Alpowa were obtained from the USDA-ARS National Small Grains Collection through GRIN-Global (https://npgsweb.ars-grin.gov/gringlobal/search). Avocet *Yr* differential lines were provided by Dr. Peng Zhang (University of Sydney). Durum wheat cv. Kronos and bread wheat cv. Ruta were used for introgression of *Yr84* into cultivated wheat backgrounds. Kronos, CDC Precision (durum wheat), and CDC Landmark (bread wheat) were used for F_2_ populations development. KSY_10-64 is an F_3_ *Yr84* tetraploid introgression line obtained from PI 487260 × Kronos (KSY_10 population), while RSY_2-2-1-2 is a hexaploid BC_2_F_3_ *Yr84* introgression line developed from a series of crosses and backcrosses for PI 487260 × Ruta.

Ten *Pst* races/isolates from different countries were used in the current study to test phenotypic responses of PI 487260: Israel — race 38E134; USA — races PST-130 (PSTv-69) and Pst-21 (provided by Dr. Xianming Chen, USDA); Canada — races W001, W020, W043, W056, W034, W049, W057 (provided by Dr. Randy Kutcher, University of Saskatchewan). Virulence profiles of all used *Pst* races are presented in Supplementary Table 5. *Pst* race W001 was selected for all the subsequent phenotypic tests described here, including BSA-Seq, F_2_, F_3_, and introgression screenings.

### Stripe rust assessment

Fresh *Pst* urediniospores were used to evaluate phenotypic responses of wheat plants to inoculation with stripe rust. The spores were considered fresh if they were collected within two weeks prior to experiments. If not used for inoculation directly after harvesting, the spores were dried in a glass desiccator filled with silica gel desiccant (VWR, Canada, catalog number CAAA44389) and kept at 4 °C prior to inoculation. Urediniospores were suspended in mineral oil (VWR, Canada, catalog number 470301) and applied at the two-leaf plant developmental stage (seedling inoculation) using an air brush compressor and air brush kit (Mastercraft, Canada, Toronto). Inoculated plants were allowed to dry in room conditions for 1.5–2 h, then placed inside a dew tent (100% humidity) in a chamber at 10 °C for 16 h in the dark, followed by 8 h of light. Next, the plants were transferred to a growth chamber (70% humidity) at 15 °C with a light intensity of 150–170 µmol m^−2^ s^−1^ for 16 h, followed by 8 h at 10 °C in darkness until plants were rated. Stripe rust severity was evaluated at 14 days post inoculation (dpi) using a 0 to 9 infection type (IT) scale^59^ with the following interpretation of the results: IT = 0–3 resistance response, IT = 4–6 moderate resistance response, IT = 7–9 susceptible response. Pictures of phenotypic responses were captured  from relevant experiments using binocular microscope Nikon SMZ1500 with Nikon C-W10xA/22 lenses, and Nikon Coolpix 8400 digital camera with Nikon Coolpix MDC Lens. Pictures were processed consistently for brightness/contrast using standard PowerPoint Picture Editing Tools.

### *Pst* spore concentration experiments

F_2_ plants from the KSY_R1 population developed from a PI 487260 × Kronos cross were inoculated with *Pst* W001 following the same procedures as described above. The solutions with spores were prepared as series of dilutions to achieve gradual changes in spore concentration. The following spore concentrations, measured using hemacytometer (Bright-Line, Hausser Scientific, Horshan, PA, USA), were used in the different treatments: Low—~624,000 spores/ml, Medium—~1,533,000 spores/ml, High—~3,059,000 spores/ml. Plants from all three treatments (81–88 plants per treatment) were inoculated at the same time with the different spore concentration solutions, and kept under the same conditions with 20–30 cm distance between plants from different treatments to prevent their contact after inoculation. Stripe rust severity was scored at 19 days post inoculation using the 0 to 9 IT scale^59^.

### Whole-genome resequencing

Twenty-six of the most resistant plants (IT = 1–4) and 26 of the susceptible plants (IT = 9) were selected after phenotyping of 92 F_2_ plants from the KSY_10 population (PI 487260 × Kronos) to form resistant and susceptible bulks, respectively. The corresponding parent was added to each bulk: PI 487260 to the resistant bulk, and Kronos to the susceptible bulk. Illumina DNA prep libraries were prepared individually for each genotype in the resistant and susceptible bulks (*n* = 27/bulk). All libraries were uniquely indexed and pooled into two bulk libraries for sequencing across two lanes each of an Illumina NovaSeq 6000 S4 flow cell.

### BSA-Seq analysis

The BSA-Seq analysis was performed using the QTL-seq algorithm^27^. Raw Illumina reads from each genotype were trimmed using Trimmomatic-0.32 and aligned to the wild emmer wheat Zavitan reference assembly^24^ with Novoalign version V4.03.01 at default parameters. Read alignments from the resistant and susceptible genotypes were concatenated to form two separate BAM files using samtools version 1.11. BAM files were then used as an input for the QTL-seq algorithm (https://github.com/YuSugihara/QTL-seq; version 2.2.2) to calculate the SNP-index for all variants between the bulks and the reference genome assembly using default parameters (except *w* = 20,000). The SNP-index is the ratio of the total number of aligned reads to the number of reads supporting a variant at a genomic locus and ranges between 0 to 1. SNP indices were then averaged over a window of 20,000 bp (with a step size of 100 kbp) to reduce potential noise from single variants resulting from mis-assembled genomic regions or spurious alignments (Supplementary Data 2). The ΔSNP-index was calculated by subtracting SNP-index of R-bulk from SNP-index of S-bulk. To define the confidence intervals of the SNP-index under the null hypothesis (no QTL), QTL-seq randomly subsamples individuals from each bulk and defines the allelic distribution at a certain read depth. This process was repeated 10,000 times for each read depth to define the coverage-adjusted confidence intervals (99 and 95%) (Supplementary Figs. 1 and 2).

### High-throughput DNA marker development and genetic map construction

The *Ku_c1312_1194*, *BS00110121*, and *Tdurum_contig44861_1253* markers from a 90 K SNP array^28^ were mapped to the Zavitan reference assembly to confirm their location on the chromosome arm 1BS within the BSA-Seq enrichment interval and then converted to KASP markers. Following the results of the first screen of the 92 F_2_ plants from the KSY_10 population with the three 90 K markers, SNPs between the resistant and susceptible bulks in the narrowed *Yr84* region were developed as KASP markers. The selection of SNPs was done iteratively and based on the results of intermediate screens used for haplotype-based analysis that guided selection of additional SNPs for KASP marker development. This procedure yielded the *usw310*-*usw323* markers (Supplementary Table 6). The KASP genotyping assays were performed following LGC Biosearch Technologies’ KASP genotyping manual (www.biosearchtech.com) on the CFX384 Touch Real-Time PCR Detection System (BioRad, Hercules, CA, US). The genetic map was constructed using QTL IciMapping V4.1 software^60^ with visualization in Mapchart V2.3 software^61^. Genetic distances in centiMorgans (cM) were converted from recombination fractions using the Kosambi function^62^.

### Dissection of the physical region of *Yr84* and candidate gene analysis

The region of interest in the Zavitan genome assembly^24^ was defined based on flanking markers that showed the closest recombination events. Sequences of all annotated genes within this interval were extracted and their function in resistance responses was predicted based on the analysis of their protein structure defined using the on-line NCBI tool Search for Conserved Domains (https://www.ncbi.nlm.nih.gov/Structure/cdd/wrpsb.cgi). The extracted Zavitan genes were BLAST in the Svevo genome assembly^23^ and annotation to find corresponding genes on chromosome 1B. To account for possible assembly errors, the genes with best hits from unassigned contigs were considered as orthologs if there was no corresponding hit from chromosome 1B. As a result of this search, the 3.37 Mb interval corresponding to location of *Yr84* in Svevo was defined and additional annotated genes residing within this region were extracted and functionally characterized in the same way as genes from the Zavitan assembly.

### Statistics and reproducibility

Statistical analyses of phenotypic data were performed in the R software environment version 4.0.2^63^. Stripe rust data were first transformed using Ordered Quantile (ORQ) normalization transformation in bestNormalize package 1.8.2^64^ to ensure residual normality and subsequent analyses were completed using both sets of data. The original dataset was used for estimation of descriptive statistics and the construction of boxplots, while a transformed dataset was used for analysis of variance (ANOVA) analysis and for multiple pairwise comparisons. Mixed-model ANOVA was performed using the PROC MIXED procedure of SAS v9.4. Genotypes, populations, or spore concentrations were considered fixed effects. The Tukey–Kramer’s post hoc test in the package rstatix (https://cloud.r-project.org/package=rstatix) was used to perform multiple pairwise comparisons. Visualization of boxplots with *p*-values obtained from the post hoc test was performed with the ggpubr package (https://CRAN.R-project.org/package=ggpubr).

### Reporting summary

Further information on research design is available in the Nature Research Reporting Summary linked to this article.

## Supplementary information

Supplementary Material

Description of Additional Supplementary Files

Supplementary Data 1-4

Reporting Summary

## Data Availability

The sequencing data is uploaded to National Center for Biotechnology Information (NCBI) under BioProject number PRJNA822617. All remaining data are available in the main text or the supplementary materials.
