# Supplementary material for: Discovery of stripe rust resistance with incomplete dominance in wild emmer wheat using bulked segregant analysis sequencing
Source: Commun Biol. 2022 Aug 17;5:826. doi: 10.1038/s42003-022-03773-3 (PMC9386016; doi:10.1038/s42003-022-03773-3)
Supplement: Supplementary file 1 — Supplementary Material [file 42003_2022_3773_MOESM1_ESM.pdf]

Supplementary Materials for

Discovery of stripe rust resistance with incomplete dominance in wild emmer wheat using bulked segregant analysis sequencing

Valentyna Klymiuk[1], Harmeet Singh Chawla[1], Krystalee Wiebe[1], Jennifer Ens[1], Andrii Fatiukha[1], Liubov Govta[2,3], Tzion Fahima[2,3], Curtis J. Pozniak[1*]

*Corresponding author. Email: curtis.pozniak@usask.ca

This PDF file includes:

Supplementary Figures 1 to 4
Supplementary Tables 1 to 6

Other Supplementary Materials for this manuscript include the following:

Supplementary Data 1 to 4

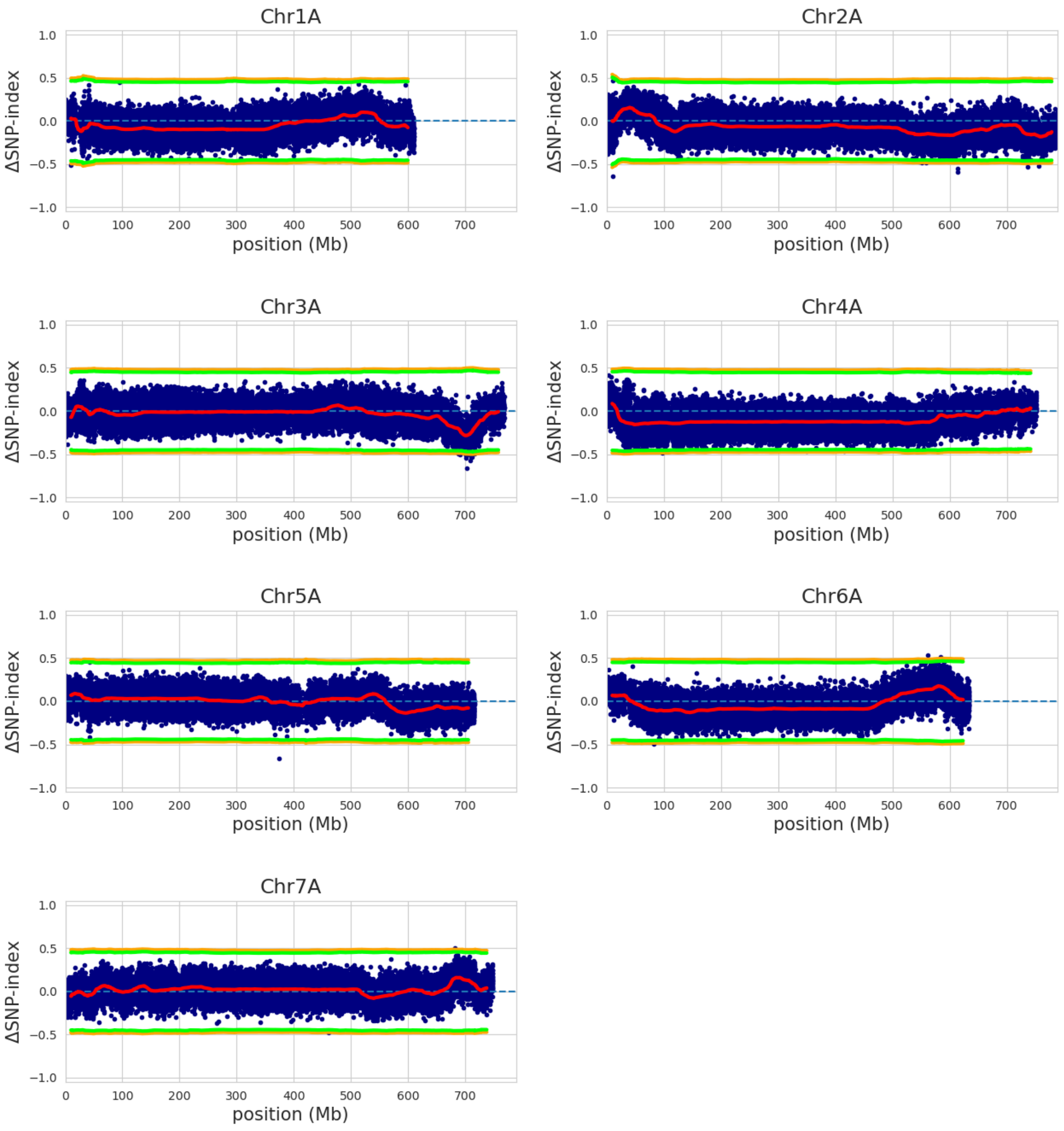

Supplementary Figure 1.

Graphical representation of ΔSNP-index across the seven wheat "A" genome chromosomes. Each plot represents the physical position (Mbp) in the Zavitan WEW_v2.0 reference assembly with ΔSNP-index on the Y-axis. Each blue dot corresponds to a SNP between the DNA bulks. The red line indicates the mean chromosomal ΔSNP-index. Orange and green lines represent coverage-adjusted confidence intervals of simulated ΔSNP-index at 99% and 95% respectively. Based on the mean chromosomal ΔSNP-index, a peak of enrichment was identified on chromosome 3A, but it did not pass the adjusted significance threshold.

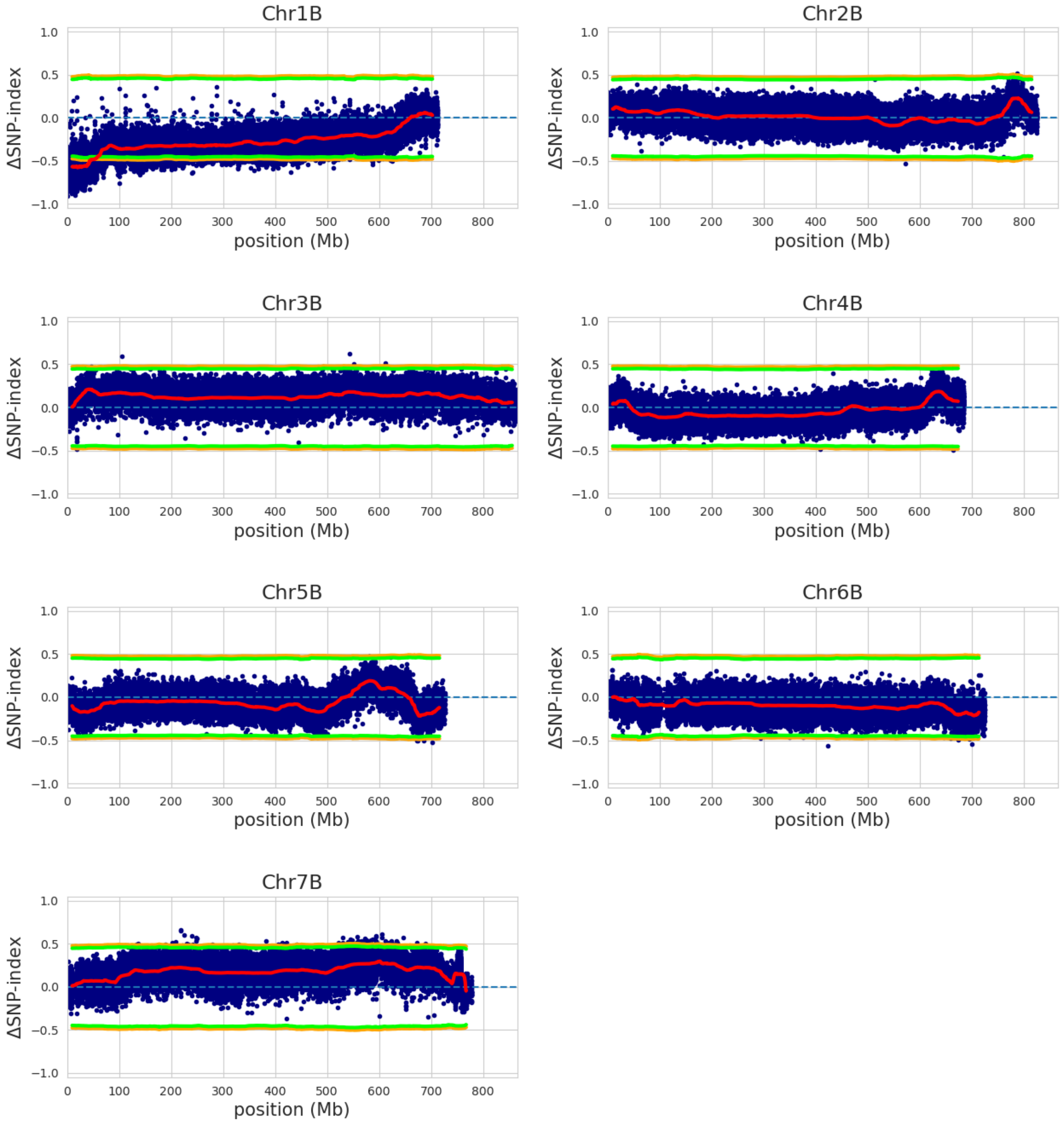

Supplementary Figure 2.

Graphical representation of ΔSNP-index across the seven wheat “B” genome chromosomes. Each plot represents the physical position (Mbp) in the Zavitan WEW_v2.0 reference assembly with ΔSNP-index on the Y-axis. Each blue dot corresponds to a SNP between the DNA bulks. The red line indicates the mean chromosomal ΔSNP-index. Orange and green lines represent coverage-adjusted confidence intervals of simulated ΔSNP-index at 99% and 95% respectively. BSA-Seq analysis showed clear enrichment in the 0-52.7 Mb region of chromosome 1B. Based on the mean chromosomal ΔSNP-index, peaks of enrichment were identified on chromosomes 2B, 4B and 5B, but these did not pass the adjusted significance threshold.

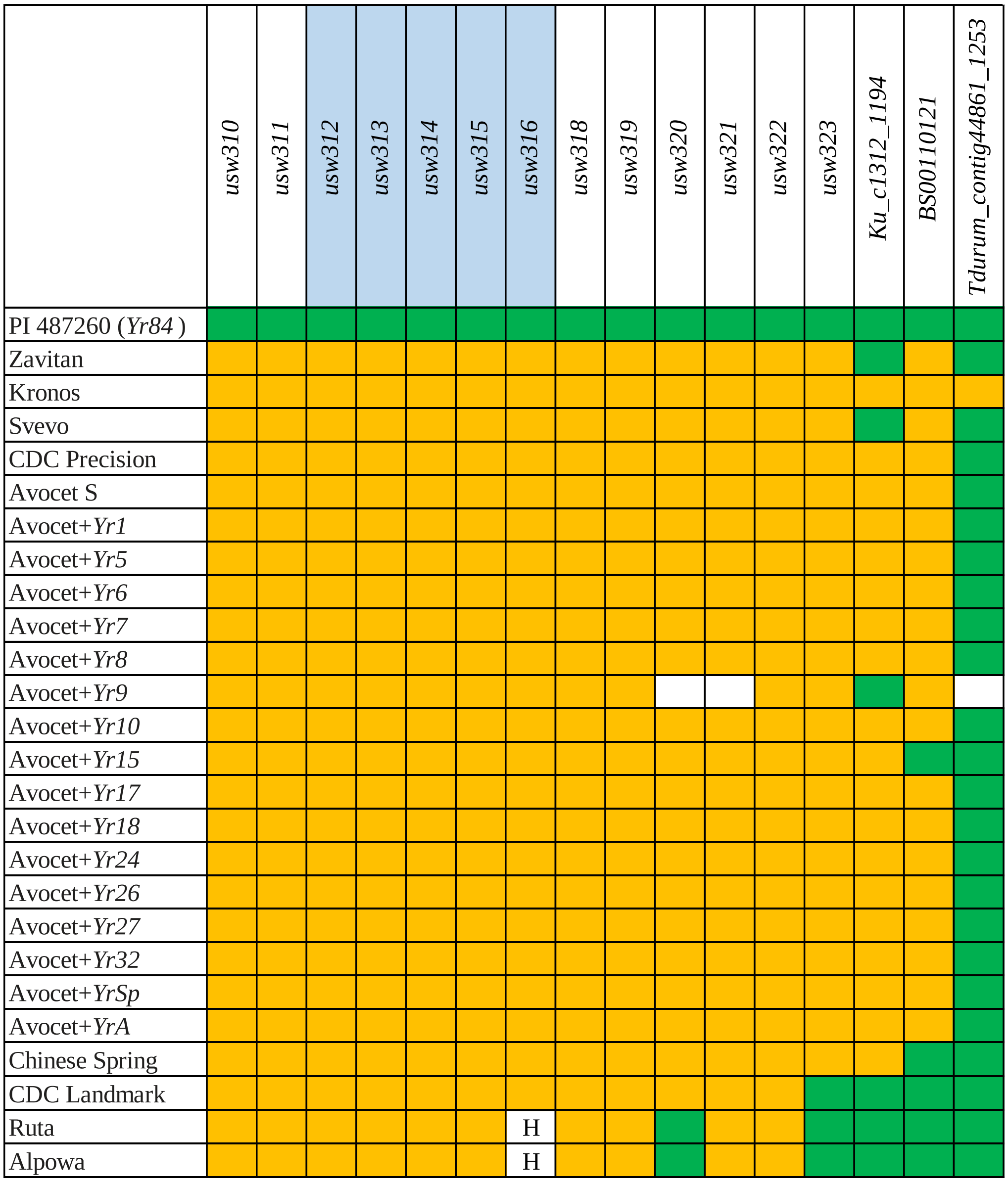

Supplementary Figure 3.

Comparison of haplotypes of several wheat lines with PI 487260 haplotype in *Yr84* region. Alleles are marked as following: green = identical to allele of the *Yr84* carrier PI 487260; yellow = KASP marker alternative allele, H = heterozygous; white – missing data. Species/ploidy relations are as following: tetraploid *T. turgidum* ssp. *dicoccoides* – PI 487260 and Zavitan; tetraploid *T. turgidum* ssp. *durum* – Kronos, Svevo, CDC Precision; hexaploid *T. aestivum* – Avocet S and *Yr* differential lines, Chinese Spring, CDC Landmark, Ruta, Alpowa. PI 487260 (*Yr84*), Avocet+*Yr1*, Avocet+*Yr5*, Avocet+*Yr10*, Avocet+*Yr15*, Avocet+*Yr24*, Avocet+*Yr26*, Avocet+*Yr32*, and Avocet+*YrSp* are resistant, Alpowa (*YrAlp*) was not tested, and the remaining genotypes are susceptible in response to seedling inoculation with *Pst* race W001. The five *Yr84* co-segregating markers are marked in blue.

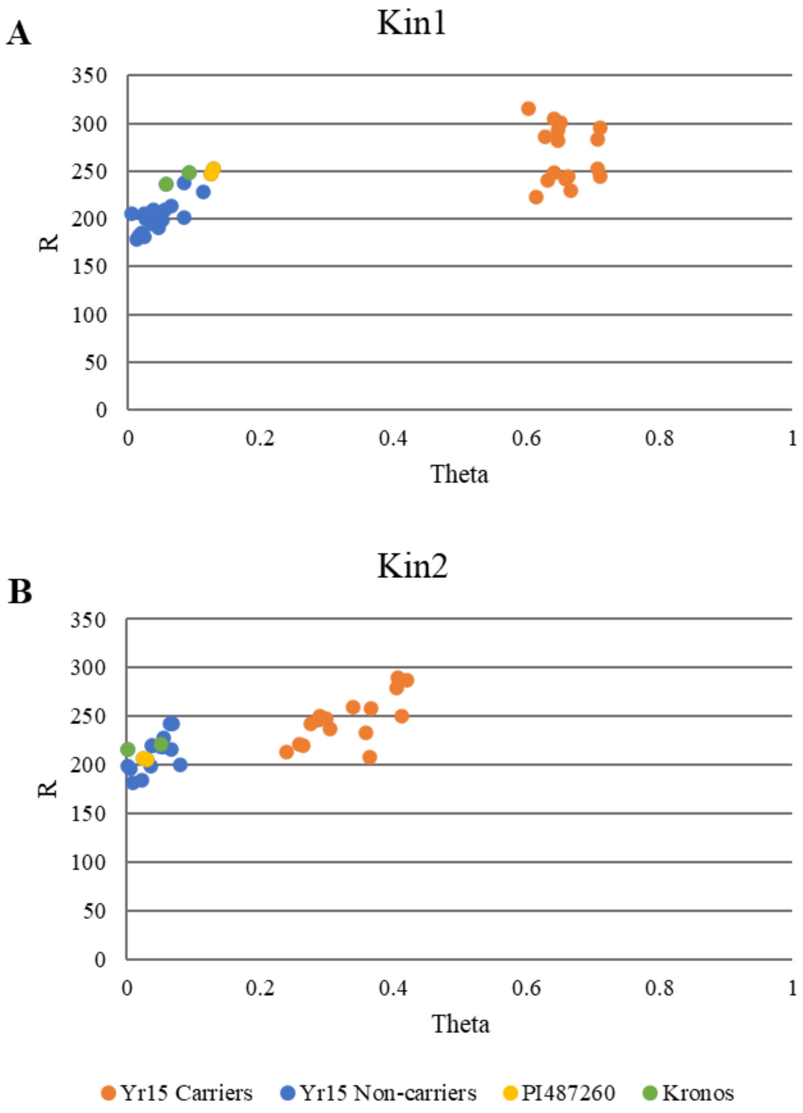

Supplementary Figure 4.

Amplification of *wtk1* (*Yr15* non-functional allele) for PI 487260 with A) Kin1 and B) Kin2 KASP *Yr15* functional markers[1]. *Yr15* carriers (*Wtk1*) are the lines G25, Avocet+*Yr15*, B9, 280-2, Excalibur+*Yr15*; while *Yr15* non-carriers (*wtk1*) are Zavitan, Chinese Spring, D447, Avocet S according to Klymiuk et al.[2].

Supplementary Table 1.

Yr84 candidate genes from Zavitan and Svevo genome annotations. Genes encoding for domains known to be involved in disease resistance responses are marked in bold; genes reported only for Zavitan assembly are marked in underlined; genes reported only for Svevo assembly are marked in italic.

**Zavitan assembly**				**Svevo assembly**			
**Gene**	**Chromosome**	**Start position**	**End position**	**Gene**	**Chromosome**	**Start position**	**End position**
TRIDC1BG001810	1B	9657221	9658930	TRITD0Uv1G100440	unassigned	305643022	305644954
TRIDC1BG001820	1B	9662447	9664099	TRITD0Uv1G100430	unassigned	305638012	305641477
TRIDC1BG001830	1B	9666416	9669055	TRITD0Uv1G003590	unassigned	7365296	7365838
**TRIDC1BG001840**	1B	9670106	9671745				
TRIDC1BG001890	1B	9722489	9722641	TRITD1Bv1G008290	1B	19410963	19411436
TRIDC1BG001900	1B	9788433	9789432	TRITD1Bv1G002150	1B	4908222	4909656
**TRIDC1BG001910**	1B	9791715	9793079	**TRITD1Bv1G008280**	1B	19376362	19378002
TRIDC1BG001920	1B	9807240	9809090				
**TRIDC1BG001850**	1B	9809856	9811400	**TRITD1Bv1G002460**	1B	5782916	5783350
**TRIDC0UG001320**	1B	9811098	9811402	**TRITD1Bv1G002140**	1B	4905408	4907606
				TRITD1Bv1G002480	1B	5821164	5827603
				TRITD1Bv1G002490	1B	5828377	5834804
				TRITD1Bv1G002640	1B	6365034	6365734
**TRIDC1BG001930**	1B	9922661	9924616	**TRITD1Bv1G002670**	1B	6400020	6509358
				TRITD1Bv1G002760	1B	6596054	6596497
**TRIDC1BG001940**	1B	9925480	9926709	**TRITD1Bv1G002530**	1B	6019827	6020306
**TRIDC1BG001950**	1B	9938048	9938358	**TRITD1Bv1G002800**	1B	6660174	6660703
				TRITD1Bv1G002780	1B	6638172	6638687
				TRITD1Bv1G002810	1B	6723074	6724354
				TRITD1Bv1G002830	1B	6740241	6741213
TRIDC1BG001970	1B	9948301	9949395	TRITD1Bv1G008140	1B	19410433	19410900
TRIDC1BG001980	1B	10000140	10001429	TRITD1Bv1G002540	1B	6126015	6127409
TRIDC1BG001990	1B	10077729	10078503				
**TRIDC1BG002000**	1B	10132824	10133921	**TRITD1Bv1G002670**	1B	6400020	6509358
**TRIDC1BG002010**	1B	10134744	10135292	**TRITD1Bv1G002670**	1B	6507917	6509688
**TRIDC1BG002030**	1B	10399473	10400086	**TRITD1Bv1G002590**	1B	6309386	6309985
TRIDC1BG002070	1B	10465688	10466959				
**TRIDC1BG002060**	1B	10483581	10485652	**TRITD1Bv1G002670**	1B	6400020	6509358
**TRIDC1BG002080**	1B	10486355	10487896	**TRITD1Bv1G002670**	1B	6948035	6952414
				TRITD1Bv1G002880	1B	6973511	6974579
**TRIDC1BG002090**	1B	10532616	10534244				
**TRIDC1BG002100**	1B	10534461	10535903				
**TRIDC1BG002110**	1B	10536057	10536593	**TRITD1Bv1G006220**	1B	14441259	14442083
TRIDC1BG002150	1B	10594558	10595529				

Zavitan assembly				Svevo assembly			
**Gene**	**Chromosome**	**Start position**	**End position**	**Gene**	**Chromosome**	**Start position**	**End position**
**TRIDC1BG002160**	1B	10671047	10672833				
TRIDC0UG001630	1B	10725659	10725865	TRITD1Bv1G002910	1B	7115618	7117651
TRIDC0UG001640	1B	10725659	10725865	TRITD1Bv1G002910	1B	7115618	7117651
TRIDC0UG001610	1B	10755535	10756088	TRITD1Bv1G002940	1B	7138088	7140239
TRIDC0UG001570	1B	10844926	10850682	TRITD1Bv1G003010	1B	7183950	7189746
**TRIDC0UG001560**	1B	10851640	10852653	**TRITD1Bv1G003020**	1B	7190716	7191729
TRIDC0UG001550	1B	10854693	10857284	TRITD1Bv1G003030	1B	7193966	7194340
TRIDC0UG001550	1B	10854693	10857284	TRITD1Bv1G003040	1B	7194548	7196524
TRIDC0UG001540	1B	10865503	10866015	TRITD1Bv1G003060	1B	7204811	7205897
**TRIDC0UG001520**	1B	10867537	10869084	**TRITD1Bv1G003060**	1B	7204811	7208438
				TRITD1Bv1G003090	1B	7330445	7331074
**TRIDC1BG002180**	1B	11032887	11035943	**TRITD1Bv1G003110**	1B	7344330	7347575
**TRIDC1BG002190**	1B	11047307	11049713	**TRITD1Bv1G003120**	1B	7357713	7361738
TRIDC1BG002210	1B	11095915	11096246	TRITD1Bv1G003150	1B	7441922	7442263
TRIDC1BG002200	1B	11178525	11179885				
TRIDC1BG002220	1B	11188633	11195858	TRITD1Bv1G003160	1B	7453950	7460862
TRIDC1BG002230	1B	11199032	11201334	TRITD1Bv1G003170	1B	7483498	7485186
TRIDC1BG002260	1B	11205370	11205870				
TRIDC1BG002270	1B	11211587	11213098				
				TRITD1Bv1G003230	1B	7672784	7673824
				TRITD1Bv1G003240	1B	7673999	7675303
TRIDC1BG002280	1B	11228824	11230656	TRITD0Uv1G003780	unassigned	7680907	7682739
				TRITD1Bv1G003270	1B	7692789	7695028
**TRIDC1BG002290**	1B	11246308	11248683				
TRIDC1BG002300	1B	11270911	11271084	TRITD1Bv1G003190	1B	7501776	7502128
TRIDC1BG002310	1B	11275738	11276083				
				TRITD1Bv1G003280	1B	7700128	7700340
TRIDC1BG002340	1B	11425210	11427732	TRITD1Bv1G003320	1B	7785088	7787598
TRIDC1BG002350	1B	11473877	11474122	TRITD1Bv1G003380	1B	7857760	7858125
TRIDC1BG002370	1B	11476963	11481821	TRITD1Bv1G003420	1B	7868458	7874157
TRIDC1BG002380	1B	11483667	11487378	TRITD1Bv1G003430	1B	7876495	7879756
				TRITD1Bv1G003400	1B	7864285	7865587
				TRITD1Bv1G003410	1B	7867227	7868009
				TRITD1Bv1G003450	1B	7907051	7908727
				TRITD1Bv1G003490	1B	8020652	8022867
**TRIDC1BG002390**	1B	11495931	11496689	**TRITD1Bv1G003470**	1B	7931510	7947292
TRIDC1BG002400	1B	11497198	11497887				
TRIDC1BG002410	1B	11501055	11502841	TRITD1Bv1G003480	1B	7990615	7992396
TRIDC1BG002420	1B	11571371	11572619	TRITD1Bv1G003500	1B	8027933	8029122

Zavitan assembly				Svevo assembly			
**Gene**	**Chromosome**	**Start position**	**End position**	**Gene**	**Chromosome**	**Start position**	**End position**
TRIDC1BG002430	1B	11577115	11578744	TRITD1Bv1G003250	1B	7686358	7688068
TRIDC1BG002430	1B	11577115	11578744	TRITD1Bv1G003510	1B	8031423	8033034
				TRITD1Bv1G003560	1B	8070853	8071351
TRIDC1BG002460	1B	11941709	11943554	TRITD1Bv1G003700	1B	8284845	8286693
**TRIDC1BG002490**	1B	11947090	11950464	**TRITD1Bv1G003680**	1B	8226869	8229643
**TRIDC1BG002500**	1B	11988955	11991946	**TRITD1Bv1G003660**	1B	8188955	8191940

Supplementary Table 2.

Fixed effect analysis of variance for stripe rust resistance in three F_2 populations KSY, PSY and LSY.

Source	*df*	F-value	p-value	significance
genotype	2	484.44	<0.001	***
population	2	35.25	<0.001	***
genotype × population	4	21.62	<0.001	***

residual variance = 1.49[#]

[#] residual variance is statistically different from zero (P<0.001)

Supplementary Table 3.

Descriptive statistics of parents and F_2 populations for stripe rust IT scores.

Genotype	Parents		No. F_2 plants	Mean	Min	Max	SD	SE
**KSY population**	PI 487260 (IT=1)	Kronos (IT=9)						
F_2 population			92	5.4	1	9	3.1	0.3
A			13	1.1	1	2	0.3	0.1
H			52	4.6	1	9	2.2	0.3
B			27	9.0	9	9	0	0
**PSY population**	KSY_10-64 (IT=1)	Precision (IT=3)						
F_2 population			94	4.4	1	9	2.6	0.3
A			30	1.3	1	2	0.5	0.1
H			48	5.1	2	7	1.4	0.2
B			16	7.9	6	9	0.9	0.2
**LSY population**	RSY_2-2-1-2 (IT=1)	Landmark (IT=8)						
F_2 population			100	6.6	2	9	2.4	0.2
A			24	2.7	2	5	1.0	0.2
H			43	7.6	6	9	0.9	0.1
B			33	8.2	7	9	0.7	0.1
**KSY Low**	PI 487260 (IT=1)	Kronos (IT=9)						
F_2 population			86	4.7	1	9	2.7	0.3
A			15	1.1	1	2	0.3	0.1
H			56	4.6	1	8	1.7	0.2
B			15	9.0	9	9	0	0
**KSY Medium**	PI 487260 (IT=1)	Kronos (IT=9)						
F_2 population			81	4.9	1	9	3.1	0.4
A			21	1.1	1	2	0.3	0.1
H			39	4.7	1	9	1.9	0.3
B			21	9.0	9	9	0	0
**KSY High**	PI 487260 (IT=1)	Kronos (IT=9)						
F_2 population			87	5.2	1	9	3.2	0.3
A			22	1.0	1	2	0.2	0.0
H			36	4.6	2	7	1.4	0.2
B			29	9.0	9	9	0	0

SD – standard deviation of the mean; SE – standard error of the mean

Supplementary Table 4.

Fixed effect analysis of variance for stripe rust resistance in the F_2 KSY population in experiments with three different spore concentrations.

Source of variation	*df*	F-value	*p*-value	significance
genotype	2	604.68	<0.0001	***
concentration	2	0.03	0.9722	ns
genotype × concentration	4	0.02	0.9995	ns

residual variance[#] = $0.16^{ns\#}$

[#] residual variance is not statistically different from zero

Supplementary Table 5.
Virulence profiles of *Pst* races used in this study.

Name of *Pst* isolate/race	Country of collection	Virulence profile
W001	Canada	YrA, Yr2, Yr6, Yr7, Yr8, Yr9, Yr17, Yr25, Yr27, Yr28, Yr29, Yr31, YrSu
W020	Canada	YrA, Yr2, Yr6, Yr7, Yr8, Yr9, Yr10, Yr17, Yr18, Yr24, Yr26, Yr27, Yr28, Yr29, Yr31, Yr32, YrSu
W034	Canada	YrA, Yr2, Yr3, Yr6, Yr7, Yr9, Yr10, Yr17, Yr24, Yr25, Yr26, Yr27, Yr28, Yr29, Yr31, Yr32
W043	Canada	YrA, Yr2, Yr3, Yr6, Yr7, Yr8, Yr9, Yr17, Yr25, Yr27, Yr28, Yr29, Yr31, Yr32, YrSu
W049	Canada	YrA, Yr2, Yr6, Yr7, Yr8, Yr9, Yr10, Yr17, Yr24, Yr25, Yr26, Yr27, Yr28, Yr29, Yr31, Yr32, YrSu
W056	Canada	YrA, Yr1, Yr2, Yr3, Yr6, Yr7, Yr8, Yr9, Yr17, Yr25, Yr27, Yr28, Yr29, Yr31, Yr32, Yr76, YrSu
W057	Canada	YrA, Yr2, Yr3, Yr6, Yr7, Yr8, Yr9, Yr10, Yr17, Yr25, Yr27, Yr28, Yr29, Yr31, Yr32, YrSu
PST-130 (PSTv-69)	USA	Yr6, Yr7, Yr8, Yr9, Yr10, Yr17, Yr27, Yr32, Yr43, Yr44, YrExp2
Pst-21	USA	Yr1
38E134	Israel	YrA, Yr2, Yr6, Yr7, Yr9, Yr18

Virulence profiles are from published materials: W001[3], W020[4], W034[3], W043[3], W049[3], W056[3], W057[3], PST-130 (PSTv-69)[5], Pst-21[6], 38E134[7].

Supplementary Table 6.

Primer sequences for KASP markers used in this study. Forward A amplifies PI 487260 allele, while forward B amplifies Kronos allele.

Marker name	Forward A	Forward B	Common reverse
Ku_c1312_1194	TGGATATCCAAGG ACGAGTAC	TGGATATCCAAGG ACGAGTAT	AAGCACTTCCTGT GGACGATC
BS00110121	ATGCGTTGATTGGA GGCATCA	ATGCGTTGATTGGA GGCATCG	GTTGCTCACAGCA GAGATCAG
Tdurum_contig4 4861_1253	GTGGACCGGACAC AGAACTCT	GTGGACCGGACAC AGAACTCG	CAAAGTAGCATCA TAGAACAC
usw310	TCGGACAGTGTTCT TTCCAAG	TCGGACAGTGTTCT TTCCAAC	GGAAGTCATCACC TCAAGTTG
usw311	GTGGTGAAGGACAG GAACGTC	GTGGTGAAGGACAG GAACGTG	AGACCGTACTTAC CAAAGGTC
usw312	CTACATCCAAAGCT CGGGACA	CTACATCCAAAGCT CGGGACC	TCCATCAGCGGTGA GGAGCAA
usw313	GCGTTTCGGTGGTG GCGGTAC	GCGTTTCGGTGGTG GCGGTAG	AGGCAGGGGAAG ATGTACCTC
usw314	CAATCCTTGGAGA AGCTCACA	CAATCCTTGGAGA AGCTCACC	CAGAGAGCAATGA TTCCTCAT
usw315	ATGAGAAGCCAACT TCAAGGG	ATGAGAAGCCAAC TTCAAGGA	CATGTAGCCATAT TATTGTCC
usw316	TGCAAGTGCTCCAA AGGCTAT	TGCAAGTGCTCCAA AGGCTAC	AGCACGGAAACTT ACTGGTGC
usw317	CTCCTCCTGCTAAT GAGCTCC	CTCCTCCTGCTAAT GAGCTCT	CATCATAGGTGAC ATGTCTTA
usw318	CAGCACGGTACAC TTGACGTC	CAGCACGGTACAC TTGACGTG	AGATCCTCCTAAG AGAGTGCA
usw319	CAACAGCAATATCT ACCAACA	CAACAGCAATATCT ACCAACT	TAGTGAGGAGCTT CCTAGAGT
usw320	GGGGCTCACCCAC AACTACTA	GGGGCTCACCCAC AACTACTT	GTGTCCATCTTGA GGTCGGAC
usw321	TGGTGAGTCATGA AAGCTTTG	CGGTGAGTCATGA AAGCTTTC	AAGCATAGCCTTG TGCAGGAG
usw322	CCTTCCTTCACACC TACTGGA	CCTTCCTTCACACC TACTGGG	GAGTGTCATTTAG AGATCAGG
usw323	AGCGTCATTCATTT CAATCAG	AGCGTCATTCATTT CAATCAC	ACGAGCATCATCA CAATGGTC

Supplementary References:

1. Klymiuk, V., Fatiukha, A. & Fahima, T. Wheat tandem kinases provide insights on disease-resistance gene flow and host–parasite co-evolution. *Plant J.* **98**, (2019).
2. Klymiuk, V. et al. Cloning of the wheat *Yr15* resistance gene sheds light on the plant tandem kinase-pseudokinase family. *Nat. Commun*. **9**, (2018).
3. Brar G.S., Ali S., Qutob D., Ambrose S., Lou K., Maclachlan R., Pozniak C.J., Fu Y.B., Sharpe A.G., Kutcher, H.R. Genome re-sequencing and simple sequence repeat markers reveal the existence of divergent lineages in the Canadian *Puccinia striiformis* f. sp. *tritici* population with extensive DNA methylation. *Environmental microbiology* **20**(4) (2018), doi.org/10.1111/1462-2920.14067.
4. Brar G.S., Population structure of *Puccinia striiformis* f. sp. *tritici*, the cause of wheat stripe rust, in western Canada (Doctoral dissertation, University of Saskatchewan).
5. Wan A., Wang X., Kang Z., Chen X., Variability of the stripe rust pathogen. In: Chen X, Kang Z, editors. Stripe Rust. Dordrecht: Springer (2017).
6. Cantu D., Segovia V., MacLean D. et al., Genome analyses of the wheat yellow (stripe) rust pathogen *Puccinia striiformis* f. sp. *tritici* reveal polymorphic and haustorial expressed secreted proteins as candidate effectors. *BMC Genomics* **14**, 270 (2013), https://doi.org/10.1186/1471-2164-14-270.
7. Yahyaoui A.H., Hakim M.S., Naimi M.E., Rbeiz N., Evolution of physiologic races and virulence of *Puccinia striiformis* on wheat in Syria and Lebanon. *Plant Disease,* **86**(5), 499-504 (2002).
